# Prediction of preoperative intrathoracic adhesions for ipsilateral reoperations: sliding lung sign

**DOI:** 10.1186/s13019-022-01844-4

**Published:** 2022-05-04

**Authors:** Gaetana Messina, Mary Bove, Antonio Noro, Giorgia Opromolla, Giovanni Natale, Francesco Leone, Vincenzo Di Filippo, Beatrice Leonardi, Mario Martone, Mario Pirozzi, Marianna Caterino, Sergio Facchini, Alessia Zotta, Giovanni Vicidomini, Mario Santini, Alfonso Fiorelli, Della Corte Carminia, Fortunato Ciardiello, Morena Fasano

**Affiliations:** 1 Thoracic Surgery Unit, Università degli Studi della Campania “Luigi Vanvitelli”, Naples, Campania Italy; 2 Oncology, Department of Precision Medicine, Università della Campania “L. Vanvitelli”, Naples, Campania Italy

**Keywords:** Ultrasound, Adherence, Sliding

## Abstract

**Introduction:**

Video-assisted thoracic surgery (VATS) for ipsilateral reoperations is controversial, because after the first surgical intervention, pleural adhesions occur frequently in the thoracic cavity and/or chest wall. This study assessed the usefulness of preoperative ultrasonography to reduce the incidence of lung injury at the time of the initial port insertion during secondary ipsilateral VATS.

**Materials and methods:**

This was a retrospective, single-center study. Nine patients who underwent thoracic surgery at Vanvitelli Hospitalfrom September 2019 to February 2022, were scheduled for a second VATS surgeryon ipsilateral lung, because of inconclusive intraoperative histologic examination. All nine patients underwent preoperative ultrasonography to assess the possible presence of pleural adhesions. We evaluated the lung sliding, since the presence of pleural adhesions does not permit to appreciate it.

**Statistical analysis:**

Hard severe adhesions were observed in all nine patients without sliding lung sign (specificity 100%). In this series, the sensitivity, PPV, and NPV of the sliding lung sign were 93%, 100% and 94% respectively.

**Results:**

The presence of the lung respiratory changes can be evaluated as the “sliding lung sign” by chest ultrasonography; we believe that the sliding lung sign might also predict intrathoracic adhesion.

**Conclusions:**

Preoperative detection of pleural adhesions using transthoracic ultrasonography was useful for ipsilateral secondary pulmonary resection patients undergoing VATS. Using preoperative ultrasonography can improve the safety and feasibility of placing the initial port in VATS.

## Introduction

Video-assisted thoracic surgery (VATS), during the last several decades, is widely used in the diagnosis and treatment of pulmonary pathologies and pleural adhesions [1, 2], and it has been validated as the favored technique for almost all thoracic surgical interventions [3–5].

The most popular technical contraindications for VATS for pulmonary resection are incomplete interlobar fissures, perivascular and/or peribronchial fibrosis, previous chemotherapy and/or radiotherapy, tumors larger than 5 cm, chest wall involvement, centrally located tumor, advanced age, severe comorbidity, severe chronic obstructive pulmonary disease (COPD), emphysema and severe pleural adhesions.

Video-assisted thoracic surgery (VATS) for ipsilateral reoperations is controversial, even for experienced surgeons, because after the first surgical intervention, pleural adhesions occur frequently in the thoracic cavity and/or chest wall.

Dense pleural adhesion can reduce the working space for the surgeon, leading to increased greater risks of complications, operative time and organ injury [6, 7].

Several authors [8, 9] have reported that almost 80% of ipsilateral reoperation patients had severe intrathoracic adhesions. The initial insertion port can lead to lung injury because of the blinded intrathoracic area during VATS.

Today ultrasonography is a modern, easy and accessible method and preoperative transthoracic ultrasonography is useful for detecting pleural adhesions in patients scheduled to undergo thoracotomy [10, 11]. Therefore, we presumed that preoperative evaluations of pleural adhesions are beneficial before surgical intervention. This study assessed the usefulness of preoperative ultrasonography to reduce the incidence of lung injury at the time of the initial port insertion during secondary ipsilateral VATS.

Prediction of intrathoracic adhesions is important to plan the surgical approach and to implement appropriate safety management [12].

The aim of this study was to determine the sensitivity, specificity and usefulness of ultrasonography in the diagnosis of preoperative lung adhesion in patients for ipsilateral reoperations and thus determine the false positive and false positive rates.

## Materials and methods

This was a prospective, single-center study. Nine patients who underwent thoracic surgery at Vanvitelli Hospital from September 2019 to February 2022were enrolled for VATS reoperations of the ipsilateral lung, because of inconclusive intraoperative histologic examination during the first surgery. All patients gave a written informed consent for the surgical treatment and were aware that all information could be anonymously used for scientific purpose only.

Chest ultrasound sonography was performed at our patient before surgery. The presence of the sliding lung sign was determined using B-mode imaging on each intercostal space of the chest. The evaluations in the upper side were difficult due to the narrower intercostal space and the scapula. Chest ultrasound sonography was mainly performed by the chief surgeon, who evaluated all data.

The study was conducted in compliance with the principles of the Declaration of Helsinki and written informed consent was obtained from all participants during preoperative communication.The protocol was approved by the Ethics Committee of the University of ‘Luigi Vanvitelli’ of Naples (n.280).

All nine patients underwent preoperative ultrasonography to assess the possible presence of pleural adhesions.

The day before VATS, we evaluated systematically with ultrasound the dorsal, lateral, and anterior chest wall to detect pleural adhesions. A linear-type (7.5 MHz) and a convex-type (3.5 MHz) ultrasound probe was used. During the ultrasonography, the patient was asked to breathe deeply, measurements in the upper side were performed within the observable range.

**Fig. 1:**
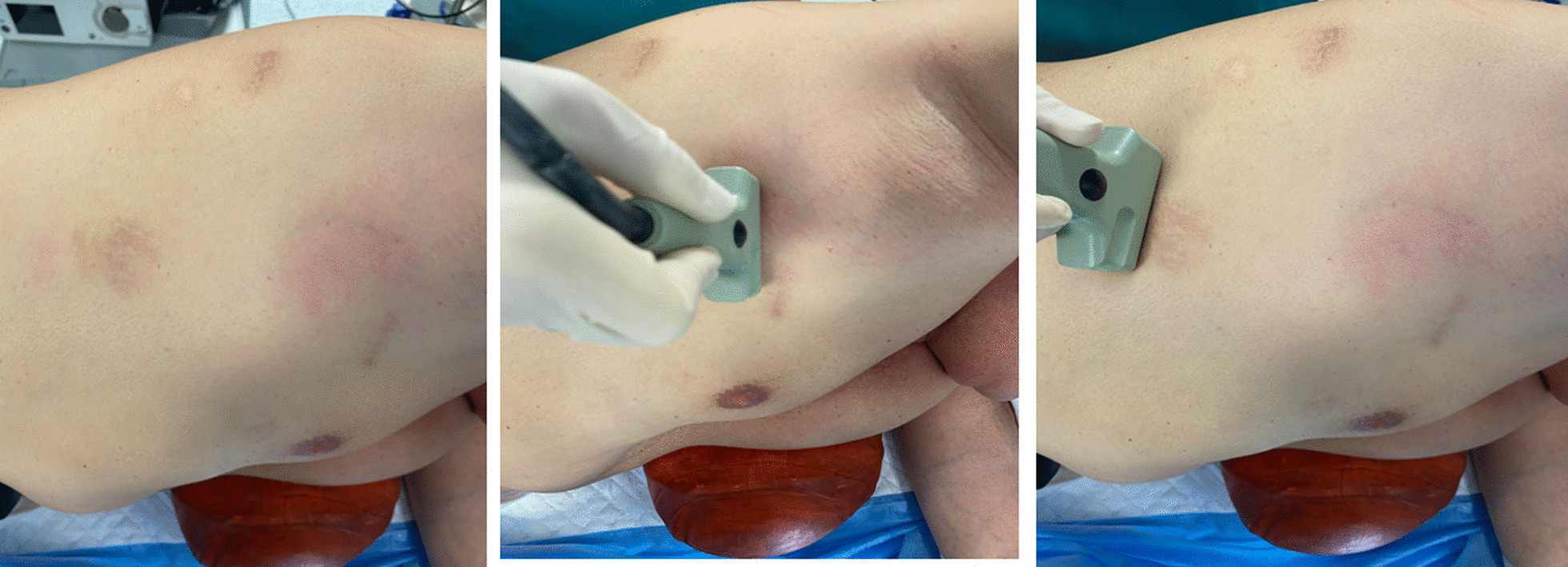
Chest ultrasonography was performed the day of the procedure, the patient was maintained in general anesthesia using single-lung ventilation with a double-lumen endotracheal tube, in the lateral decubitus position on the operating table

In addition, chest ultrasonography was performed the day of the procedure: the patient was in lateral decubitus position on the operating table, under general anesthesia intubated with a double-lumen endotracheal tube in order to obtain single-lung ventilation (Fig. 1).

VATS lobectomy employed is the tri-portal approach according to Hansen et al. [13]. It consists in two 1–1.5 cm lower access incisions for two thoracoscopic ports located in the 7th or 8th intercostal space, in the posterior and anterior axillary line respectively, and a 4–5 cm port incision, placed in the 4th intercostal space, in the anterior axillary for a utility incision.

**Fig. 2:**
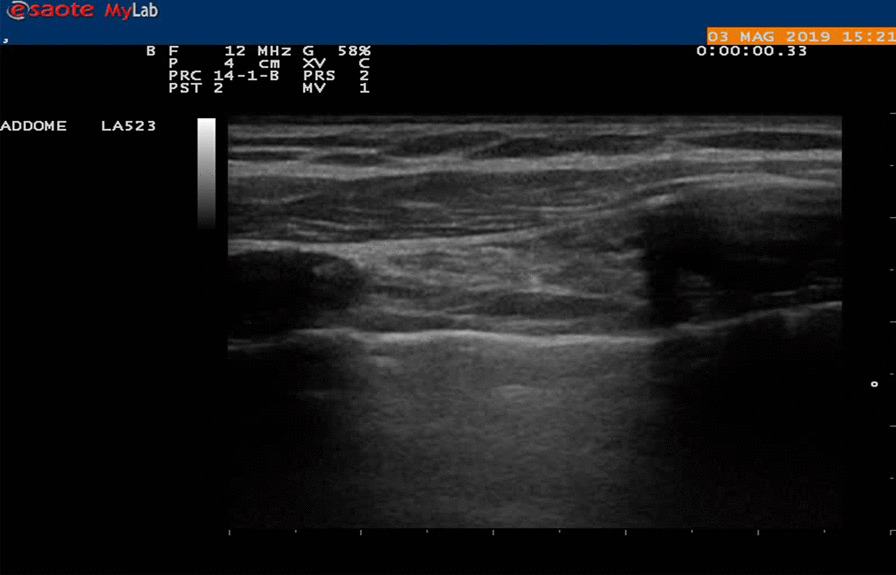
Sliding is an echographic phenomenon produced during the normal respiratory cycle the visceral pleura slides on the parietal pleura

**Fig. 3:**
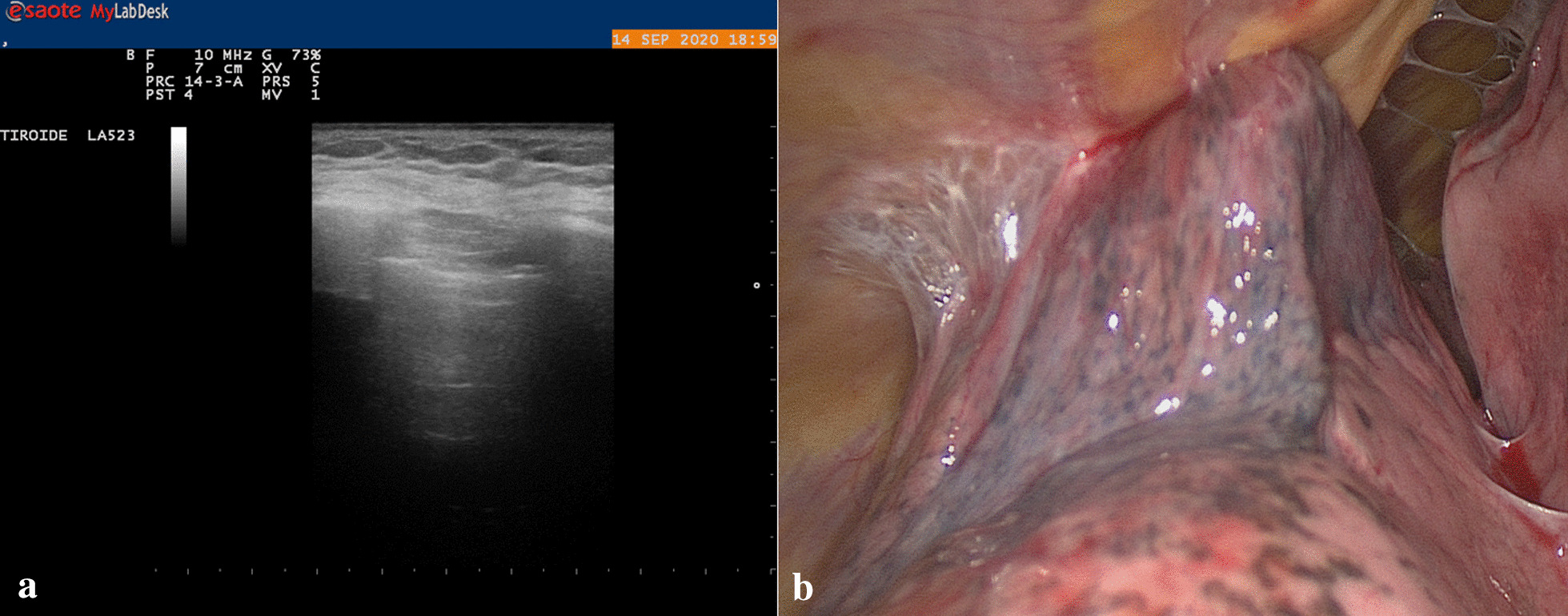
**a** Absence Sliding. **b** Adherence is in the site where the port was previously introduced during the first ipsilateral intervention

**Fig. 4:**
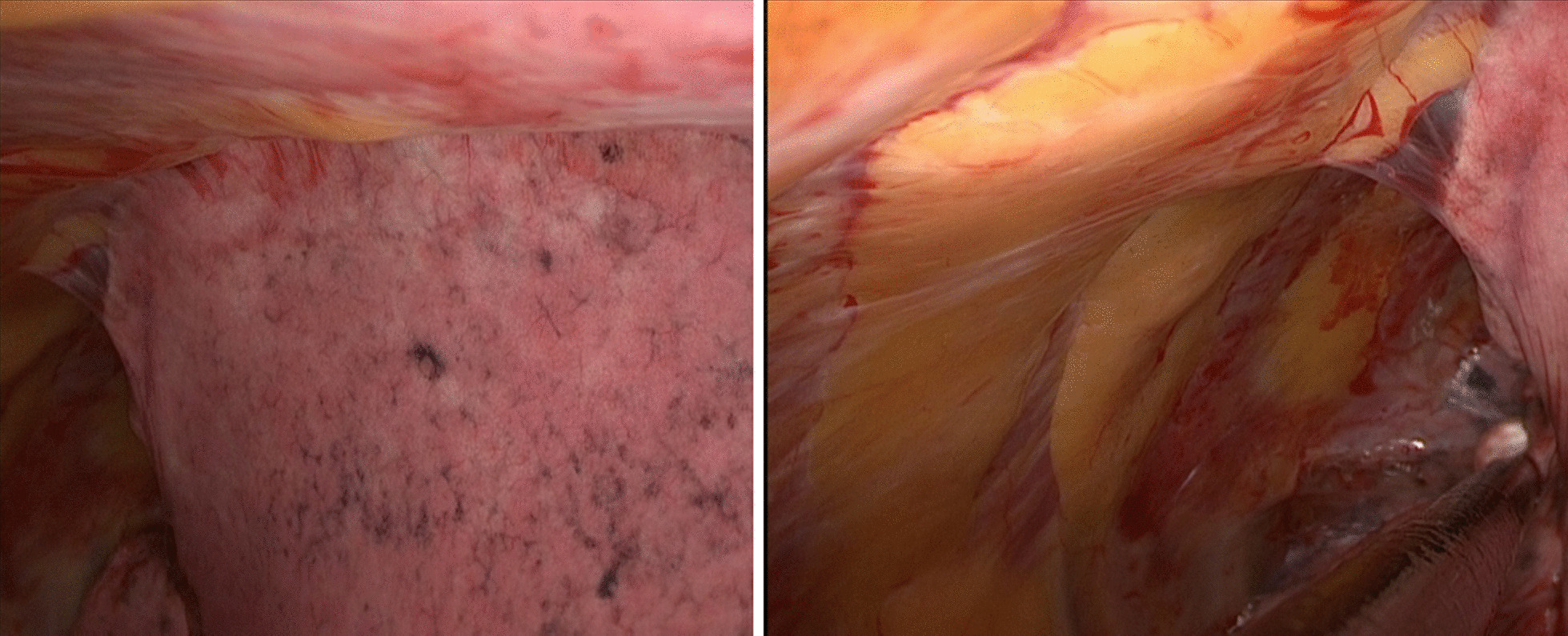
Pleural adhesion

All patients were examined with ultrasound study using ultrasound machine with convex (3.5 MHz) and linear (7.5 MHz) transducers, in the lateral position with the seventh or eighth intercostal space of the midaxillary line undergoing bipulmonary ventilation. We evaluated the lung sliding, an echographic phenomenon produced during the normal respiratory cycle, the visceral pleura slides on the parietal pleura (Fig. 2) [14]. Presence of pleural adherence does not permit the observer to appreciate this sign (Fig. 3) [15]. To fix the better entry site, we searched for areas with a good echographic sliding sign and a normal appearance of the chest wall and pleural line, when the appropriate echographic aspects were not present, we examined other adjacent areas to avoid pleural adhesion. (Fig. 4) After having identified with chest sonography the ideal entry site, we performed local anesthesia with Mepivacaine (200 mg) and after making a small skin incision, we slowly introduced curved blunt-point scissors into the chest wall as far as the pleural space, then a blunt-point trocar was carefully introduced and air spontaneously allowed to enter the pleural space with consequent lung collapse; therefore when pleural sliding sign is present, pleural adhesions are absent and the lung can collapse when the trocar is inserted into the pleural cavity. Applying this method, we always were able to avoid pleural adherence.

The lung sliding sign can distinguish between dense adhesions and non-dense adhesions. If there were dense adhesions, then the lung would not slide with breathing.

Severe intrathoracic adhesion was defined as the need for adhesiolysis requiring more than 30 min.

The VATS technique used for ipsilateral reoperation did not differ considerably from that used for routine lung cancer cases, except in the evaluation of adhesions using preoperative ultrasonography.

Three surgeons performed the surgeries; complicated surgeries for lobectomy were performed by the chief surgeon. A 24-Fr chest tube was inserted at the end of the procedure. Prolonged air leakage was defined as an air leak lasting more than 5 days postoperatively. The chest tube duration was measured in days.

### Statistical analysis

The type of adhesion was divided into hard or soft adhesion.

Hard severe adhesions were observed in all nine patients without sliding lung sign (specificity 100%). In this series, the sensitivity, PPV, and NPV of the sliding lung sign were 93%, 100% and 94% respectively. There was only one false-negative result, both of which were soft adhesions [Table 1].

**Table 1:** Specificity the sensitivity, PPV, and NPV of the sliding lung sign were: 100%, 93%, 100%, and 94%, respectively

SENSITIVITY	SPECIFICITY	PPV	NPV
93%	100%	100%	94%

There was only one false-negative result, both of which were soft adhesions

## Results

The present retrospective observational and single-center study observed nine patients (6 male and 3 female) who underwent thoracic surgery at Vanvitelli Hospital from September 2019 to February 2022. These patients underwent a second VATS to re-operate the ipsilateral lung, because of intraoperative inconclusive histological examination during the first surgery.

**Fig. 5:**
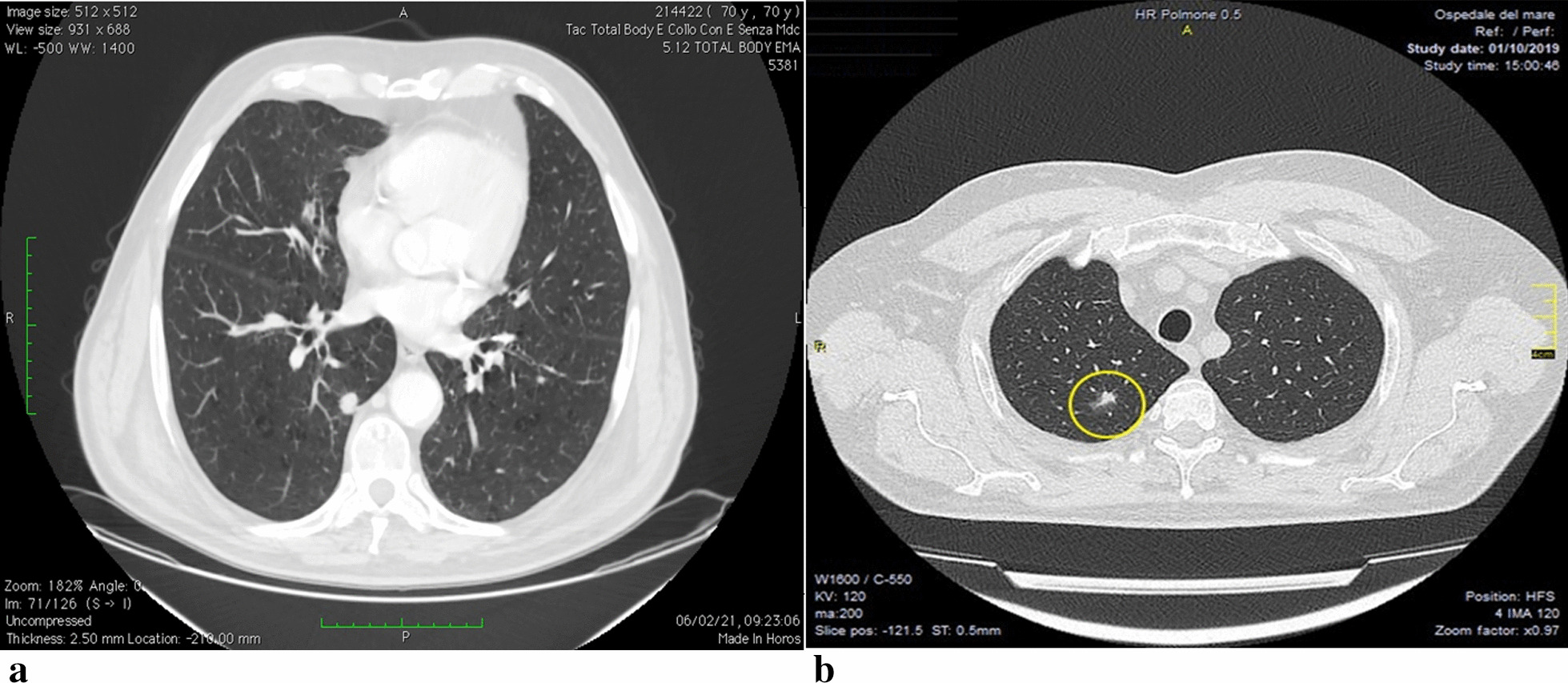
**a** Nodule in right lower lung lobe, pet positive, in a patient with a history of breast cancer. **b** Nodule in upper lobe right lung, pet positive, in a patient with a history of prostate cancer

2 patients underwent resection of a nodule in right lower lobe (Fig. 5a), and 1 patient underwent resection of nodule in left lower lobe (3 female, aged about 72 and 67 years), PET positive, in a patient with a history of breast cancer; the intraoperative histological examination was inconclusive due to differential diagnosis between lung adenocarcinoma and mammary carcinoma, therefore the lobectomy was waiting for definitive differential diagnosis; the definitive histological examination confirms the diagnosis of primary lung cancer, therefore after 20 days the patients underwent right lower lobectomy and left lower lobectomy;

3 patients underwent resection nodule in right lower lobe and 1 patient underwent resection nodule in left upper lobe (4 men aged about 77 and 78 years), (Fig. 5b) PET positive, with a history of prostate cancer, the intraoperative histological examination was inconclusive due to the differential diagnosis between lung adenocarcinoma and prostate cancer, therefore the lobectomy was waiting for definitive differential diagnosis; the patients were reoperated after about 23 days and they were underwent a left lower lobectomy and a one patient was underwent left upper lobectomy;

2 patient (2 men, aged about 74 and 76 years) with nodule in the upper left lung, PET positive, with a history of renal cell carcinoma, intraoperative histological examination was inconclusive due to the differential diagnosis between lung adenocarcinoma and renal cell carcinoma, therefore the lobectomy was waiting for definitive differential diagnosis; the patient were reoperated after about 18 days and they underwent a left upper lobectomy [16].

The following patient, ultrasound, and surgical characteristics and follow-up parameters were recorded: age, past medical history, smoking history, gender, history of exposure to asbestos, interstitial pneumonia, preoperative respiratory function measured by spirometry, procedure type, surgical approach (complete VATS vs. thoracotomy), intraoperative blood loss, duration of chest tube, duration of surgery, complications (prolonged air leak defined as an air leak lasting longer than 5 days, pneumonia, delirium, arrhythmia, chylothorax, acute respiratory distress syndrome,), and duration of postoperative hospitalization [Table 2].

**Table 2:** Patients’ background

Variable	N = 5
Age (years)	73.8 [67–78]
Gender	6 male; 3 female
*Smoking history*	
Heavy smokers	4
No smoker	1
Asbestos history	1
Left-sided disease	1
Restrictive lung disease	0
Obstructive lung disease	3
Interstitial lung disease	1
Previous breast cancer	3
Previous prostate cancer	4
Previous kidney cancer	2
Surgical approach	9 VATS
Intraoperative blood loss (ml)	95 [120 ± 35]
Duration of chest tube (days)	3 [2–7]
Duration of surgery (min)	145 ± 38
Postoperative hospitalization (days)	4 [3–8]
Persistent air leaks (> 5 days)	1

All nine patients presented adhesions at the initial port during VATS. Only one adhesion was not detected with preoperative ultrasound and the other adhesions were found using preoperative ultrasonography that was performed for all patients. Among these nine cases, we were able to place the initial port avoiding the adhesions during VATS.

**Fig. 6:**
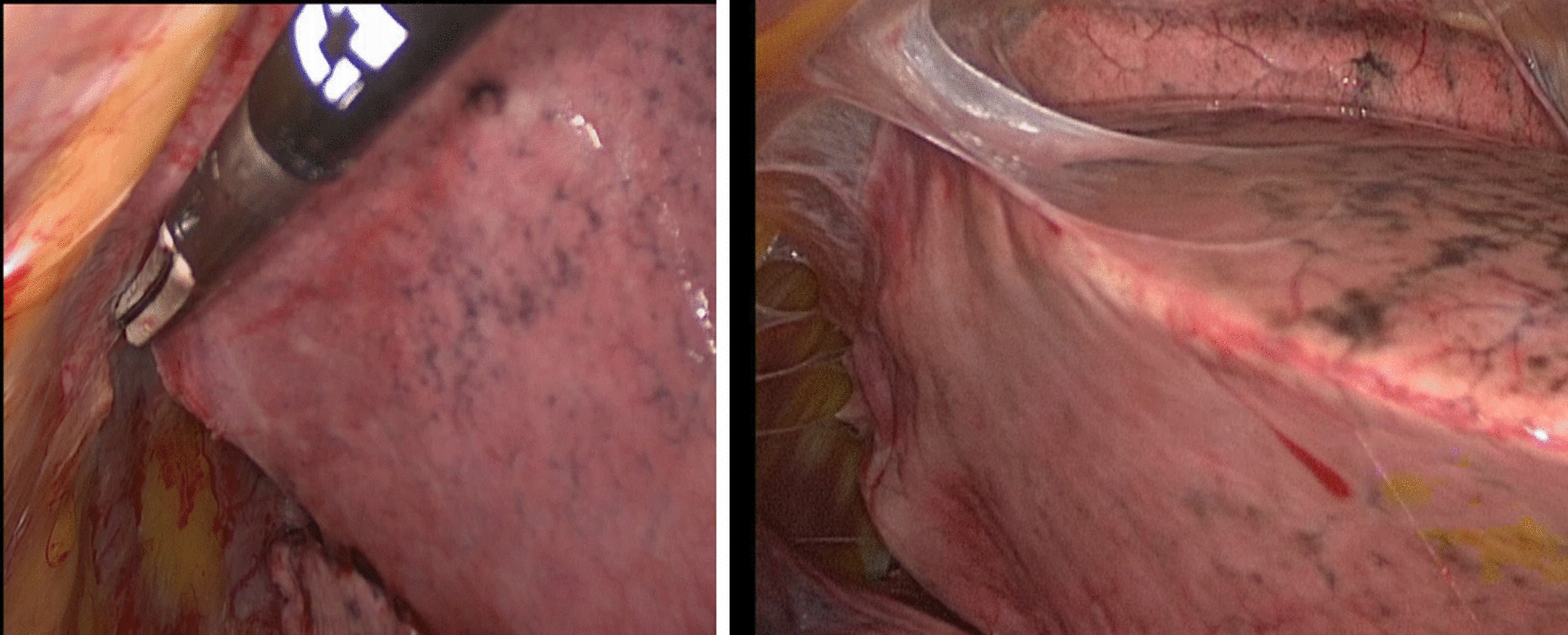
Only one adhesion was not detected with preoperative ultrasound; it was removed easily, without lung injury or massive bleeding

We compared the adhesions detected with transthoracic ultrasound with the ones detected in VATS. Visceral slide sonography detected the following adhesions: one false-negative adhesions, thirteen true-positive adhesions, zero false-positive adhesions, an eighteen true- negative adhesions [17] [Table 3]. Undetected adhesions were removed easily, without lung injury or massive bleeding, because they were loose and could be removed quickly, luckily there was no conversion from VATS to open thoracotomy (Fig. 6). The surgical procedure performed was lobectomy, with the mean operative time of 145 ± 38 min. The mean intraoperative bleeding volume was 95 ml (120 ± 35 ml). The mean chest tube duration was 3 days.

**Table 3:** Visceral slide sonography detected the following adhesions: 1 false-negative adhesions, 13 true-positive adhesions, 0 false-positive adhesions, 18 true-negative adhesions

FALSE-NEGATIVE	TRUE-POSITIVE	FALSE-POSITIVE	TRUE-NEGATIVE
1	13	0	18

Fortunately, there were no complications, such as, myocardial infarction, cerebral infarction, postoperative bleeding, arrhythmia, bronchial fistula, or pneumonia for any patients. One patient had prolonged (defined as more than 5 days) air leakage for 7 days.

Furthermore, there were no conversions from VATS to open thoracotomy [18].

## Discussion

For thoracic surgeons, Video-Assisted Thoracoscopic surgery (VATS) is an important diagnostic tool that is widely used in the diagnosis and treatment of pulmonary pathologies and pleural adhesions [1, 2].

Unexpected intrathoracic adhesions are relatively uncommon complications of thoracic surgery.

The VATS technique used for ipsilateral reoperation did not differ considerably from that used for routine lung cancer cases, except in the evaluation of adhesions. Severe intrathoracic adhesions were significantly associated with more bleeding, longer operative time, thoracotomy conversion, increased postoperative complications, and longer postoperative hospital stays, the presence of adhesions might be a prognostic factor for prolonged postoperative air leakage.

Loose adhesions and dense adhesions were considered blunt ablations that could or could not be removed easily.

If adhesions were expected on preoperative ultrasonography, the initial port was placed at a location where ultrasonography showed no adhesions. We checked for adhesions and determined their locations during VATS.

Pleural adhesions occur frequently in pulmonary vessels and bronchial and pulmonary surgical stumps in the thoracic cavity and/or chest wall because of secondary intrathoracic changes after the first surgical intervention. Several authors, have reported that surgeons failed to perform complete VATS; there was a 6.5–23% rate of conversion from complete VATS to open surgery [19, 20]. Chen et al. [8] have reported that almost 78% of ipsilateral reoperation patients had dense intrathoracic adhesions. Approximately 24% of conversions were related to intense adhesions and massive intraoperative bleeding [15, 16].

Two studies conducted so far have shown that ultrasonography can be used to determine pre-surgical pleural adhesions [21, 22]. In a study by Kolecki et al., specificity and sensitivity of ultrasound were found to be 92%, and 90% respectively [23]. Mason and Tateishi in the two separate studies in this case, found a sensitivity of 72% and 75%, and a specificity of 71% and 93% respectively [21, 24]. In another study, which evaluated pleural adhesion by sonography, its sensitivity of sonography was 75% and the specificity was 93% [25].

The sliding lung sign is a sonographic finding that reflects the back-and-forth sliding of the visceral and parietal pleura past one another during respiration. The presence of the lung respiratory changes can be evaluated as the “sliding lung sign” by chest ultrasonography [26]; we believe that the sliding lung sign might also predict intrathoracic adhesion. Several studies have reported the utility of ultrasonography for determining intrathoracic adhesions [27, 28] and several reports of patients who required reoperations for ipsilateral pulmonary lesions have demonstrated that patients in the VATS group had shorter hospital stay and fewer complications than patients in the open thoracotomy, VATS is feasible and safe for selected patients [8, 29, 30].

Furthermore, for inexperienced surgeons, VATS in the presence of dense pleural adhesions can reduce the working space, leading to increased greater risks of complications, operative time and unexpected massive bleeding and/or lung injury [6, 7]. We speculated that preoperative evaluation of pleural adhesions is beneficial before surgical intervention. Predicting severe intrathoracic adhesions before surgery has many advantages: it enables appropriate decision-making with respect to surgical approach, appropriate time management of the surgery, safe installation of the first port and assessment of the risk of complications, it may improve operative times and patient safety [12].

Therefore, it is critically important that all medical staff, as well as surgeons and assistants, is adequately prepared, alleviating the stress on the surgeon and other staff, it may improve operative times; if a patient has too much severe adhesions, the appropriate treatment would be to use a minimal thoracotomy approach. We presumed that preoperative evaluations of pleural adhesions are beneficial before surgery.

Therefore, preoperative chest ultrasonography might be a helpful method to reduce complications during VATS for ipsilateral secondary lung resection patients. Moreover, use of preoperative ultrasonography could decrease factors of prolonged postoperative air leakage due to pleural adhesions.

It enables appropriate decision-making with respect to surgical approach, safe installation of the first port, appropriate time management of the surgery, and assessment of the risk of complications. Therefore, it is critically important that all medical staff as well as surgeons and assistants be adequately prepared.

The understanding of the sliding lung sign is easy once you see it, and everyone can immediately practice and evaluate it quite precisely [31]. However, evaluation of adhesions of the hilum, mediastinum, and apex are difficult using ultrasonography. Transparietal ultrasound does not allow to evaluate adhesions in consideration of the mediastinal and diaphragmatic pleura, because of the high acoustic impedance of the expanded lung.

## Conclusions

Today ultrasonography is a modern, easy and accessible method.

Preoperative transthoracic ultrasonography was useful for detecting pleural adhesions in patients who had undergone thoracic surgical intervention [10, 32]. Therefore, before pulmonary resection, preoperative ultrasonography was used to evaluate the feasibility of providing safe thoracoscopic access without lung injury and/or unexpected massive bleeding, thereby facilitating VATS [11]. Preoperative ultrasonography was used to determine the existence of adhesions in patients undergoing reoperation. Especially for ipsilateral secondary VATS, insertion of the initial port can be the most crucial point of leading to lung injury because of the blinded intrathoracic area. If adhesions were visible, adhesions can be removed carefully during VATS, then pulmonary resection can be performed safely, without lung injury and massive bleeding. Ultrasonography is a simple non-invasive procedure that provides real-time and immediate results and itmight become a more important examination for clinicians. Furthermore, ultrasonography offers other advantages; free from radiation hazards and relatively cost-effective [33]. Ultrasonography is easy to learn and use, portable, and accurate when examining the pleural space, it has allowed for safer pleural procedures, including that of VATS. In conclusion, preoperative detection of pleural adhesions using transthoracic ultrasonography was useful for ipsilateral secondary pulmonary resection patients undergoing VATS. Using preoperative ultrasonography can improve the safety and feasibility of placing the initial port in VATS.

The understanding of the sliding lung sign is easy once you see it, and everyone can immediately practice and evaluate it quite precisely [34].

We demonstrated that preoperative detection of pleural adhesions using transthoracic ultrasonography could provide safe thoracoscopic access among ipsilateral secondary thoracic surgery patients without unexpected massive bleeding and/or lung injury by enabling the placement of the initial port. This method can also be used for robotic surgery, because also this approach needs the intoduction of ports; however further data is needed to corroborate our data.

## Data Availability

The authorsdeclarethat the data supporting the findings of this study are availablewithin the article and itssupplementary information files.
